# Evaluation for causal effects of socioeconomic traits on risk of female genital prolapse (FGP): a multivariable Mendelian randomization analysis

**DOI:** 10.1186/s12920-023-01560-5

**Published:** 2023-06-09

**Authors:** Wei Zhang, Jing Ge, Zhaohui Qu, Wenjuan Wu, Hua Lei, Huiling Pan, Honggu Chen

**Affiliations:** 1 Department of Critical Care Medicine, Wuhan Jinyintan Hospital, Tongji Medical College, Huazhong University of Science and Technology, Wuhan, 430023 Hubei Province People’s Republic of China; 2 Department of Tuberculosis, Wuhan Jinyintan Hospital, Tongji Medical College of Huazhong, University of Science and Technology, Wuhan, 430023 Hubei Province People’s Republic of China; 3 Department of Orthopedics, the Affiliated Hospital of Jiangsu University, Zhenjiang, 212000 Jiangsu Province People’s Republic of China

**Keywords:** Educational attainment, Female genital prolapse, Multivariable Mendelian randomization, Causality

## Abstract

**Background:**

Although observational studies have established some socioeconomic traits to be independent risk factors for pelvic organ prolapse (POP), they can not infer causality since they are easily biased by confounding factors and reverse causality. Moreover, it remains ambiguous which one or several of socioeconomic traits play predominant roles in the associations with POP risk. Mendelian randomization (MR) overcomes these biases and can even determine one or several socioeconomic traits predominantly accounting for the associations.

**Objective:**

We conducted a multivariable Mendelian randomization (MVMR) analysis to disentangle whether one or more of five categories of socioeconomic traits, “age at which full-time education completed (abbreviated as “EA”)”, “job involving heavy manual or physical work (“heavy work”)”, “average total household income before tax (income)”, “Townsend deprivation index at recruitment (TDI)”, and “leisure/social activities” exerted independent and predominant effects on POP risk.

**Methods:**

We first screened single-nucleotide polymorphisms (SNPs) as proxies for five individual socioeconomic traits and female genital prolapse (FGP, approximate surrogate for POP due to no GWASs for POP) to conduct Univariable Mendelian randomization (UVMR) analyses to estimate causal associations of five socioeconomic traits with FGP risk using IVW method as major analysis. Additionally, we conducted heterogeneity, pleiotropy, and sensitivity analysis to assess the robustness of our results. Then, we harvested a combination of SNPs as an integrated proxy for the five socioeconomic traits to perform a MVMR analysis based on IVW MVMR model.

**Results:**

UVMR analyses based on IVW method identified causal effect of EA (OR 0.759, 95%CI 0.629–0.916, *p* = 0.004), but denied that of the other five traits on FGP risk (all *p* > 0.05). Heterogeneity analyses, pleiotropy analyses, “leave-one-out” sensitivity analyses and MR-PRESSO adjustments did not detect heterogeneity, pleiotropic effects, or result fluctuation by outlying SNPs in the effect estimates of six socioeconomic traits on FGP risk (all *p* > 0.05). Further, MVMR analyses determined a predominant role of EA playing in the associations of socioeconomic traits with FGP risk based on both MVMR Model 1 (OR 0.842, 95%CI 0.744–0.953, *p* = 0.006) and Model 2 (OR 0.857, 95%CI 0.759–0.967, *p* = 0.012).

**Conclusion:**

Our UVMR and MVMR analyses provided genetic evidence that one socioeconomic trait, lower educational attainment, is associated with risk of female genital prolapse, and even independently and predominantly accounts for the associations of socioeconomic traits with risk of female genital prolapse.

**Supplementary Information:**

The online version contains supplementary material available at 10.1186/s12920-023-01560-5.

## Introduction

Pelvic organ prolapse (POP), predominantly including uterine prolapse and vaginal prolapse (i.e., female genital prolapse, FGP), is a clinically common and distressing entity in women across all ages worldwide and has become an increasing socioeconomic problem with an estimated prevalence of 3–6% in the general population [1]. POP can significantly impact a woman’s quality of life [2, 3], causing discomfort, pain, urinary incontinence [4], and sexual dysfunction [5]. While the prevalence of POP is high, the etiology of this condition remains poorly understood. Previous observational studies have reported that such representative indicators for socioeconomic status as less education [6–9], heavy physical labor [7, 9–13] and lower income [14] are independent risk factors for higher odds of having POP *via* multivariate logistic regression analysis. However, these independent risk factors of POP have been challenged by some concurrent contradictive findings [15–18]. Furthermore, the conclusions drawn from observational studies are unable to infer causality regarding the role of socioeconomic traits in the development of FGP, since they may be confined by potential methodological limitations such as confounding and reverse causality [19, 20], which obscures the true causal relationship. Understanding the causal factors underlying POP is essential for developing effective prevention and treatment strategies. For example, identifying modifiable risk factors for POP could help to prevent the onset of the condition or slow its progression.

Mendelian randomization (MR) approach, utilizing genetic variants (GVs) as instrumental variables (IVs) for an exposure, provides an estimation for potential causal relationship between an exposure and an outcome [21, 22]. Since it uses GVs to imitate random allocation from parents to their offspring, MR can overcome the issues of confounding and reverse causality [23]. Although it is nontrivial to disentangle the causal associations of individual socioeconomic traits (such as educational attainment, heavy physical labor, income status, accommodational conditions, poverty degree, and leisure or social activities) with FGP risk, it would be also difficult to identify GVs that are solely associated with one socioeconomic trait, but not with the others, in consideration of pleiotropic effects of these GVs [24]. So, it remains ambiguous which one or several of these traits play predominant role in the causal relationship between socioeconomic traits and FGP risk.

Multivariable Mendelian randomization (MVMR), an extension approach of traditional MR method by integrating a set of SNPs in a same model, simultaneously estimates potential causal effects of multiple exposures on an outcome, in order to clarify which one or several of these exposures predominantly account for the causal effects [25, 26].

Therefore, we conducted a series of univariable Mendelian randomization analyses (UVMR) to determine whether each representative of the six socioeconomic domains, namely, educational attainment (EA), heavy physical labor, income status, accommodational conditions, poverty degree and leisure or social activities, was causally associated with FGP risk, and then a MVMR analysis to disentangle whether one or more of the six socioeconomic traits, was predominantly relevant to FGP risk.

## Materials and methods

### Study design

**Fig. 1:**
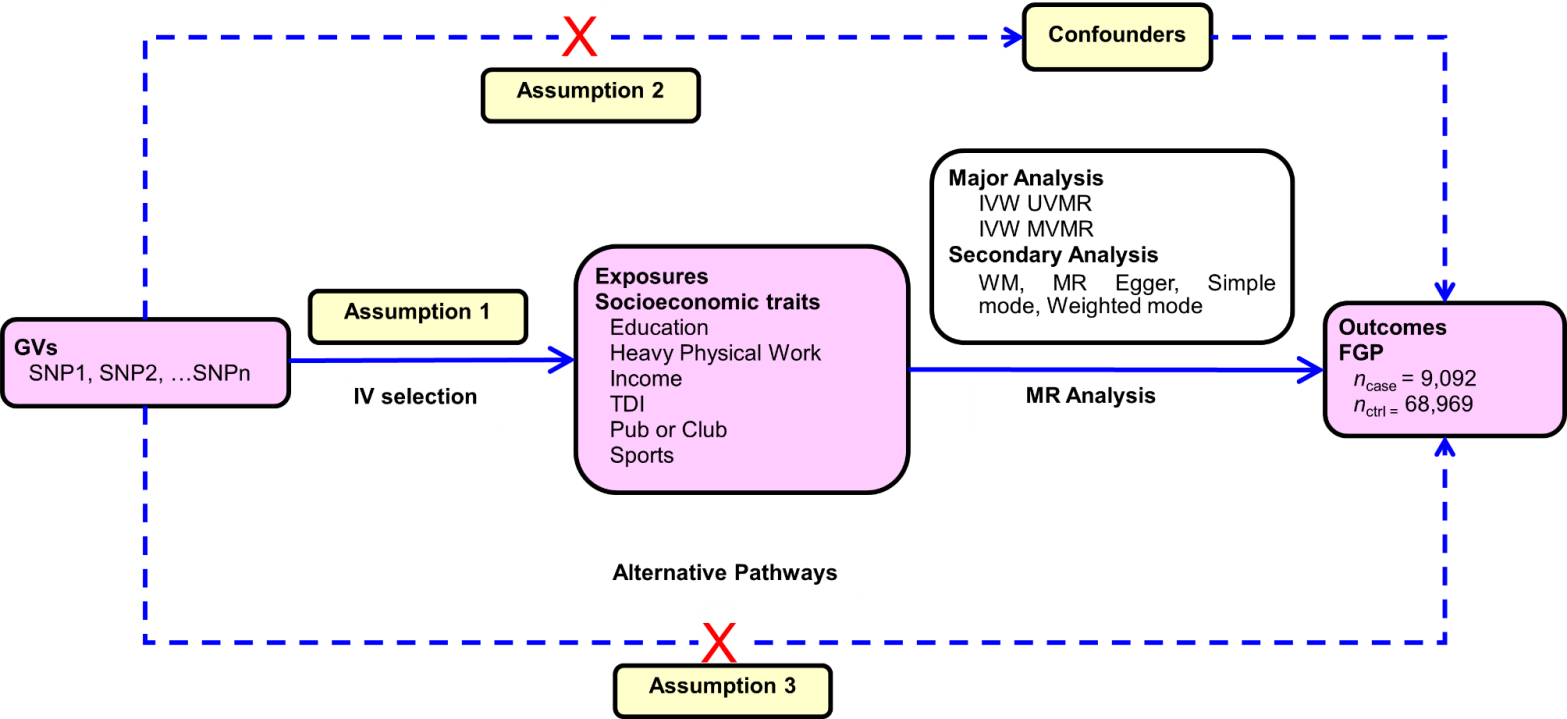
**Overview of MR rationale, design, and procedures.** There are three key assumptions for MR study. Assumption 1: the GVs selected as IVs should be strongly associated with the exposures; Assumption 2: the used GVs should not be associated with any potential confounder; Assumption 3: the GVs should influence the outcome risk merely through the exposures, not *via* any alternative pathway. **Abbreviations**: ctrl = control; FGP = female genital prolapse; GVs = genetic variants; IV = instrumental variable; IVW = inverse variance weighted; MR = Mendelian randomization; MVMR = multivariable Mendelian randomization; SNP = single-nucleotide polymorphism; TDI = Townsend deprivation index; UVMR = univariable Mendelian randomization; WM = weighted median

We first performed forward directional UVMR analyses. Then, to expel the possibility of reverse causality, we attempted to perform reverse directional UVMR analyses to examine whether genetically proxied FGP had a causal effect on each of the six categories of socioeconomic traits. An overview of the rationale, design, and procedures for our MR study are exhibited in Fig. 1.

### Data source

We extracted SNPs that were strongly associated with six categories of socioeconomic traits (EA, heavy physical work, income status, accommodational conditions, poverty degree, and leisure or social activities) from the GWASs undertaken by MRC-IEU, Neale Lab, and SSGAC Consortium. If there were more than one GWAS providing summary data for one same category of socioeconomic trait (e.g., EA), only the one GWAS (e.g., GWAS for “educational attainment” that was undertaken by SSGAC including 1,131,881 participants [27]) was chosen and retained in consideration of its largest sample size. To prevent population stratification bias, we selected SNPs and their summary statistics (β, standard error, and *p*-value) from the studies including only individuals of European ancestry [28]. We obtained summary-level data on the outcome (i.e., FGP) from a GWAS in the FinnGen Research Project (ID: finn-b-N14_FEMGENPROL; https://gwas.mrcieu.ac.uk/datasets/finn-b-N14_FEMGENPROL/), which comprising data for 16,377,670 SNPs from 78,061 participants (9,092 cases and 68,969 controls) of European ancestry. The characteristics of GWAS data for six categories of socioeconomic traits are exhibited in Table 1.

**Table 1:** Characteristics of GWAS data for six categories of socioeconomic traits

Category	Traits	Sample size	SNPs	Consortium	Link	Year
Educational attainment	Age completed full time education	307,897	9,851,867	MRC-IEU	https://gwas.mrcieu.ac.uk/datasets/ukb-b-6134/	2018
	Age completed full time education	226,899	10,894,596	Neale Lab	https://gwas.mrcieu.ac.uk/datasets//	2017
	Year ended full time education	112,569	9,851,867	MRC-IEU	https://gwas.mrcieu.ac.uk/datasets//	2018
	Qualifications: College or University degree	458,079	9,851,867	MRC-IEU	https://gwas.mrcieu.ac.uk/datasets//	2018
	Qualifications: College or University degree	334,070	10,894,596	Neale Lab	https://gwas.mrcieu.ac.uk/datasets//	2017
	Educational attainment	1,131,881	10,101,242	SSGAC	https://thessgac.com/	2018
Heavy physical work	Job involves heavy manual or physical work	263,615	9,851,867	MRC-IEU	https://gwas.mrcieu.ac.uk/datasets/ukb-b-2002/	2018
	Job involves heavy manual or physical work	190,643	10,894,596	Neale Lab	https://gwas.mrcieu.ac.uk/datasets/ukb-a-503/	2017
Income status	Average total household income before tax	397,751	9,851,867	MRC-IEU	https://gwas.mrcieu.ac.uk/datasets/ukb-b-7408/	2018
Accommodational conditions	Type of accommodation lived in: A flat, maisonette or apartment	360,088	13,586,555	Neale Lab	https://gwas.mrcieu.ac.uk/datasets/ukb-d-670_2/	2018
	Type of accommodation lived in: A house or bungalow	360,088	13,586,555	Neale Lab	https://gwas.mrcieu.ac.uk/datasets/ukb-d-670_1/	2018
	Own or rent accommodation lived in: Live in accommodation rent free	356,340	10,768,474	Neale Lab	https://gwas.mrcieu.ac.uk/datasets/ukb-d-680_6/	2018
	Own or rent accommodation lived in: Own outright (by you or someone in your household)	356,340	13,586,423	Neale Lab	https://gwas.mrcieu.ac.uk/datasets/ukb-d-680_1/	2018
	Own or rent accommodation lived in: Rent - from local authority, local council, housing association	356,340	13,586,423	Neale Lab	https://gwas.mrcieu.ac.uk/datasets/ukb-d-680_3/	2018
	Own or rent accommodation lived in: Rent - from private landlord or letting agency	356,340	13,586,423	Neale Lab	https://gwas.mrcieu.ac.uk/datasets/ukb-d-680_4/	2018
	Own or rent accommodation lived in: Own with a mortgage	356,340	13,586,423	Neale Lab	https://gwas.mrcieu.ac.uk/datasets/ukb-d-680_2/	2018
Poverty degree	Townsend deprivation index at recruitment	336,798	10,894,596	Neale Lab	https://gwas.mrcieu.ac.uk/datasets/ukb-a-44/	2017
	Townsend deprivation index at recruitment	462,464	9,851,867	MRC-IEU	https://gwas.mrcieu.ac.uk/datasets/ukb-b-10011/	2018
Leisure or social activities	Leisure/social activities: pub or social club	461,369	9,851,867	MRC-IEU	https://gwas.mrcieu.ac.uk/datasets/ukb-b-4171/	2018
	Leisure/social activities: Sports club or gym	461,369	9,851,867	MRC-IEU	https://gwas.mrcieu.ac.uk/datasets/ukb-b-4000/	2018

Abbreviations: GWAS = genome-wide association studies; MRC-IEU = The Medical Research Council Integrative Epidemiology Unit at the University of Bristol; SNP = single-nucleotide polymorphism; SSGAC = Social Science Genetic Association Consortium

### Instrumental variable selection

Valid instrumental variables are defined by three key assumptions (Fig. 1) that they associate with the exposure factors of interest (the relevance assumption); that they share no common causes with the outcome (the independence assumption); and that they do not affect the outcome except through the exposure factors (the exclusion restriction assumption). In order to meet the relevance assumption, the first of the three key assumptions, instrumental variants should be associated with the exposure factors of interest [29]. The SNPs associated with six categories of socioeconomic traits, namely, EA, heavy physical work, income status, accommodational conditions, poverty degree, and leisure or social activities, were extracted at a genome-wide significance level (*p* < 5 × 10^− 8^) from the GWAS datasets [30]. Afterwards, independent SNPs for exposures were obtained by linkage disequilibrium (LD) clumping with a threshold *r*^2^ < 0.001 and an allele distance > 10,000 kb [31]. We then extracted the SNPs and corresponding statistics from the GWAS dataset for outcome (i.e., FGP), removing the SNPs with a minor allele frequency (MAF) < 0.01 [28]. Further, we harmonized socioeconomic and FGP data by removing all palindromic SNPs [32]. In the context of socioeconomic-FGP relationship, such FGP-relevant traits or risk factors as BMI [6, 33–39], waist circumference [6, 33–35], smoking [35, 36, 40], diabetes [34], and menopausal status [36, 41, 42], are most likely to be major confounders. To fulfill the second MR assumption, we inquired for each IV and its proxy traits referring to PhenoScannerV2 database (http://www.phenoscanner.medschl.cam.ac.uk/) and discarded the SNPs surrogating for these confounding traits at a threshold of *r*^*2*^ > 0.80 [43, 44]. Accordingly, these rigorously selected SNPs were used as IVs for the following UVMR and MVMR analyses. In addition, all the removed SNPs and the reasons why they are excluded are exhibited in Supplementary Table 1. Also, we conducted reverse directional MR analyses to investigate a potential causal effect of genetically proxied FGP on the six categories of socioeconomic traits, respectively. To this end, we likewise selected SNPs that were genome-wide significant (*p* < 5 × 10^− 8^) and independently inherited (*r*^2^ < 0.001) without LD for FGP, and then extracted SNPs and corresponding statistics from the GWAS datasets for the six categories of socioeconomic traits. As shown in Table 1, we respectively identified six, two, one, seven, two, and two GWAS datasets to investigate potential causal effects of these six categories of socioeconomic traits on FGP risk by UVMR analysis. However, we only retained one trait whose GWAS dataset had the largest sample size within this socioeconomic category. Therefore, the following eight socioeconomic traits were chosen as the representatives for the six categories of socioeconomic traits: (1) EA (*n* = 1,131,881), (2) job involves heavy manual or physical work (*n* = 263,615), (3) average total household income before tax (*n* = 397,751), (4) type of accommodation lived in: a flat, maisonette or apartment (*n* = 360,088) or type of accommodation lived in: a house or bungalow (*n* = 360,088), (5) Townsend deprivation index at recruitment (*n* = 462,464), and (6) leisure/social activities: pub or social club (*n* = 461,369) or leisure/social activities: sports club or gym (*n* = 461,369).

### Instrumental strength

First, we computed the proportion of phenotypic variation that is explained by all SNPs (i.e., R^2^-values) in our MR analysis using the formula R^2^ = Σ [2 × (1 – MAF) × MAF × (β/ SD)^2^ [45, 46] where SD and β are the standard deviation and β coefficient for effect size, and MAF is the minor allele frequency for each SNP. Then, we calculated F-statistic to evaluate the instrumental strength of our SNPs for each socioeconomic trait in explaining phenotypic variation using the formula F = [(N - k − 1)/k] × [R^2^/ (1 - R^2^)] [47] where N is the sample size, k is the total number of SNPs that are selected for MR analysis, and R^2^ is the total proportion of phenotypic variation that is explained by all the SNPs in our MR analysis. A F-statistic > 10 suggests that the combined SNPs in our IVW MVMR model is a sufficiently strong instrument to explain phenotypic variation, while a F-statistic ≤ 10 implies a weak instrument [47].

### UVMR analysis

Afterwards, we undertook a series of UVMR analyses to estimate the causal associations of genetically proxied socioeconomic traits with FGP risk using five MR methods, inverse variance weighted (IVW), MR-Egger, weighted median (WM), simple mode, and weighted mode [48]. The IVW method uses a meta-analysis approach to combine the Wald ratios of the genetically causal effects of each SNP, relying on the assumption that all SNPs are valid IVs with no evidence of directional pleiotropy [28]. So, it is considered to provide an estimate with the highest power and the best precision, and is used as major analysis [24, 49]. Odd ratios (ORs) and corresponding 95% confidence intervals (CIs) were calculated for estimating causal effects of the six categories of socioeconomic traits on FGP risk. In order to account for multiple testing of the five MR methods, we used the Bonferroni correction [50]. We calculated a Bonferroni-corrected *p* threshold, by dividing 0.05 by the number of tests, which assumes each test is independent [51–54]. We considered a *p* value less than Bonferroni-corrected *p* threshold as being statistically significant [50, 55, 56], and that larger than Bonferroni-corrected *p* threshold but less than 0.05 was suggestive of statistical significance in the univariable MR analysis [50].

### Heterogeneity, pleiotropy, and sensitivity analysis

After forward and reverse directional UVMR analyses, we conducted heterogeneity, pleiotropy, and sensitivity analysis to verify whether heterogeneity and pleiotropy biased our UVMR results. First, we calculated Q-statistics and I^2^ (%)-values to quantitatively evaluate the heterogeneity level across individual SNPs [57]. Thereafter, we conducted “leave-one-out” sensitivity analyses by removing a different SNP in each iteration to clarify whether the overall MR estimates were affected by removed SNPs [58]. If one or more SNPs were detected to be responsible for drastic alteration of overall MR estimates, it or they would be removed and MR analyses be re-performed. After that, we evaluated the pleiotropy of our effect estimates with MR-Egger intercept method, using “mr_pleiotropy_test” function in R TwoSampleMR package. Furthermore, we applied the Pleiotropy RESidual Sum and Outlier (MR-PRESSO) analysis [59] to provide outlier-adjusted estimates of causal associations by removing one or more pleiotropic outlying SNPs and re-conducting MR analyses.

### MVMR analysis

Further, we used IVW MVMR method to disentangle which one or several of these socioeconomic traits predominantly accounted for the causal associations with FGP risk. Unlike UVMR analysis, MVMR analysis assumes that the IVs are strongly associated with at least one exposure, although not necessarily with each. To this end, we converged a combination of SNPs as an integrated proxy for the six categories of socioeconomic traits. Additionally, we performed feature selection for these six categories of socioeconomic traits using the “mv_lasso_feature_selection” function, and excluded one or more exposure traits with severe collinearity out of the subsequent MVMR analysis. Then, we performed MVMR analysis using “mv_multiple” function in R TwoSampleMR package. Similarly, ORs and corresponding 95% CIs were calculated for estimating causal effects. Bonferroni correction accounting for multiple testing was not tailed for MVMR analysis, since the latter has its inherent nature of mutual adjustment [24].

### Statistical power

Moreover, we determined the statistical power in estimating causal effects of socioeconomic traits on FGP risk using a webpage-based application, the mRnd power calculator (https://shiny.cnsgenomics.com/mRnd/) [60].

### Software and packages

All statistical analyses and visualization for results were performed using R statistical software (version 4.1.0, R Foundation for Statistical Computing, Vienna, Austria; https://www.R-project.org) with the “TwoSampleMR”, “LDlinkR”, and “forestplot” Packages.

## Results

### Eligible SNPs

After we removed SNPs with LD or MAF < 0.01, palindromic SNPs, and confounder-related SNPs (detailed in Supplementary Table 1), we retained 320 SNPs serving as IVs for “EA”, 15 SNPs for “job involves heavy manual or physical work”, 32 SNPs for “average total household income before tax”, one SNP for “type of accommodation lived in: a flat, maisonette or apartment”, one SNP for " type of accommodation lived in: a house or bungalow”, 13 SNPs for “Townsend deprivation index at recruitment”, ten SNPs for “leisure/social activities: pub or social club”, and seven SNPs for “leisure/social activities: sports club or gym” to perform UVMR analyses (Table 2).

**Table 2:** UVMR analyse for genetically causal associations of socioeconomic traits with FGP risk

Socioeconomic traits	nSNP	R2 (%)	F-statistic	IVW				WM				MR-Egger			Power(%)
				OR	95% CI	*p*		OR	95% CI	*p*		OR	95% CI	*p*	
Educational attainment	320	0.597	21.225	0.759	0.629–0.916	0.004		0.779	0.590–1.029	0.079		0.893	0.434–1.841	0.76	41
Job involves heavy manual or physical work	15	0.07	12.326	1.518	0.779–2.956	0.22		1.053	0.434–2.551	0.910		1.524	0.049–47.149	0.814	22
Average total household income before tax	32	0.115	14.311	1.257	0.775–2.037	0.354		1.485	0.844–2.615	0.17		1.304	0.127–13.389	0.825	32
PPRESSO adjustment	31	0.111	14.251	1.402	0.898–2.189	0.137		1.538	0.850–2.781	0.155		0.912	0.109–7.642	0.933	21
Type of accommodation lived in: a flat, maisonette or apartment	1	NA	NA	NA	NA	NA		NA	NA	NA		NA	NA	NA	NA
Type of accommodation lived in: a house or bungalow	1	NA	NA	NA	NA	NA		NA	NA	NA		NA	NA	NA	NA
Townsend deprivation index at recruitment	13	0.151	53.81	0.598	0.232–1.542	0.287		1.212	0.385–3.815	0.743		0.147	0.001–30.421	0.496	32
Leisure/social activities: pub or social club	10	0.028	12.995	0.136	0.006–3.239	0.218		0.36	0.015–8.712	0.529		0.391	1.070 × 10^− 6^–1.430 × 10^5^	0.889	33
PPRESSO adjustment	8	0.02	11.757	0.524	0.023–11.765	0.684		1.004	0.038–26.615	0.998		0.015	0.182 × 10^− 6^–1.250 × 10^3^	0.495	10
Leisure/social activities: sports club or gym	7	0.041	27.238	1.753	0.053–58.028	0.753		9.512	0.228–396.676	0.237		0.835	0.408 × 10^− 17^–1.710 × 10^17^	0.993	25

Notes: R2 represents the total proportion of phenotypic variation that is explained by SNPs in MR analyses. F-statistic denotes the total instrumental strength of the SNPs for each socioeconomic trait. An F-statistic > 10 suggests the strong instrument to explain phenotypic variation

Abbreviations: CI = confidence interval; IVW = inverse-variance weighted; MR = Mendelian randomization; OR = odds ratio; SE = standard error; nSNP = number of single-nucleotide polymorphism; WM = weighted median

### Effect estimations based on IVW UVMR

As shown in Table 2, higher EA (OR 0.759, 95%CI 0.629–0.916, *p* = 0.004) has a causal association with lower FGP risk based on IVW UVMR model. That is to say, the women possessing higher EA have approximately one-quarter less probability of incident FGP compared to their counterparts. However, the other five traits have no causal effects on odds of FGP (“Job involves heavy manual or physical work”: OR 1.518, 0.779–2.956, *p* = 0.220; “average total household income before tax”: OR 1.257, 0.775–2.037, *p* = 0.354; “Townsend deprivation index at recruitment”: OR 0.598, 0.232–1.542, *p* = 0.287; “leisure/social activities: pub or social club”: OR 0.136, 0.006–3.239, *p* = 0.218; “leisure/social activities: sports club or gym”: OR 1.753, 0.053–58.028, *p* = 0.753). The results regarding causal associations of the eight socioeconomic traits with FGP risk by UVMR analyses based on three MR methods are demonstrated in Table 2. Also, we conducted reverse directional MR analyses to investigate potential causal effect of genetically proxied FGP on each of eight socioeconomic traits respectively. After we removed SNPs with a series of procedures above, we retained eight SNPs serving as IVs for FGP to perform reverse directional UVMR analyses. Our reverse directional UVMR analyses show that genetically proxied FGP has a causal association with none of the eight socioeconomic traits based on IVW UVMR model (“EA”: OR 0.999, 95%CI 0.987–1.011, *p* = 0.810; “job involves heavy manual or physical work”: OR 0.997, 0.981–1.012, *p* = 0.659; “average total household income before tax”: OR 1.004, 0.986–1.022, *p* = 0.669; “type of accommodation lived in: a flat, maisonette or apartment”: OR 0.998, 0.995–1.002, *p* = 0.377; “type of accommodation lived in: a house or bungalow”: OR 1.002, 0.998–1.006, *p* = 0.341; “Townsend deprivation index at recruitment”: NA; “leisure/social activities: pub or social club”: OR 1.002, 0.997–1.007, *p* = 0.473; “leisure/social activities: sports club or gym”: OR 0.998, 0.991–1.005, *p* = 0.596). That is to say, our analyses expel the possibility of reverse causality between genetically proxied FGP and any of eight socioeconomic traits.

### Sensitivity, heterogeneity, and pleiotropy

**Fig. 2:**
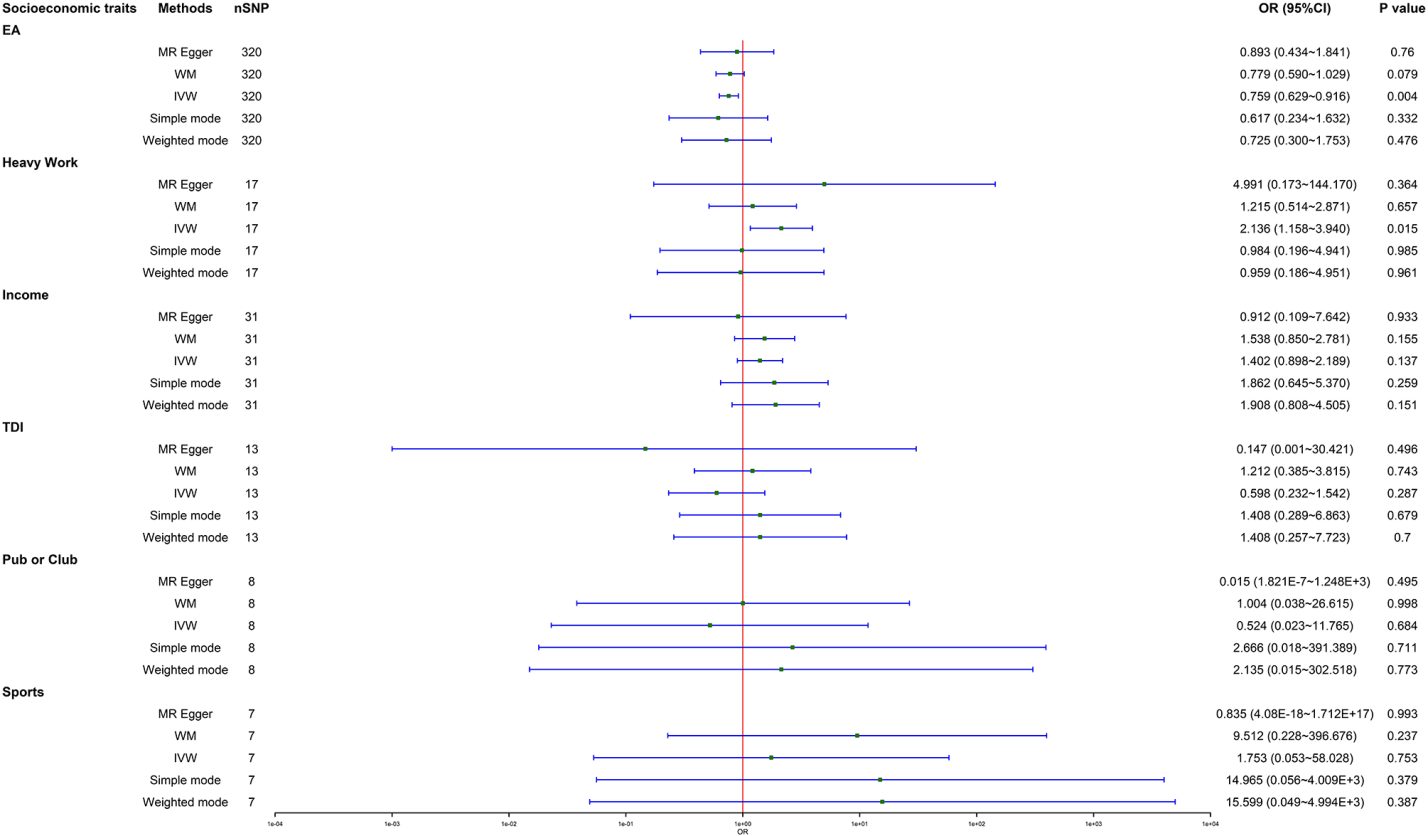
**Forrest plot for causal associations of socioeconomic traits with FGP risk based on five UVMR methods.** The forest plot suggests causal effect of genetical predisposition to lower EA on higher FGP risk based on IVW UVMR method. Inconsistently, the other five socioeconomic traits do not have any causal effect on FGP risk. **Abbreviations**: CI = confidence interval; EA = “educational attainment”; FGP = female genital prolapse; heavy physical work = “job involves heavy manual or physical work”; income = “average total household income before tax”; IVW = inverse-variance weighted; MR = Mendelian randomization; nSNP = number of single-nucleotide polymorphism; OR = odds ratio; pub or club = “leisure/social activities: pub or social club”; sports = “leisure/social activities: sports club or gym”; TDI = “Townsend deprivation index at recruitment”; UVMR = univariate Mendelian randomization; WM = weighted median

As indicated in Table 3, there exists pronounced heterogeneity regarding the effect estimates of “average total household income before tax” (Q = 47.818, I^2^ = 35.171%, *p* = 0.027) and “leisure/social activities: pub or social club” (Q = 21.437, I^2^ = 58.017%, *p* = 0.011) on FGP risk in UVMR analyses. Our “Leave-one-out” sensitivity analyses did not detect noticeable alterations in effect estimates when any one SNP was removed (Supplementary Figs. 1–6), suggesting robustness in our UVMR results. Additionally, as demonstrated in Table 3, there is no apparent pleiotropy concerning the effect estimates of all the six socioeconomic traits on FGP risk in UVMR (“EA”: Egger intercept = -0.002, SE = 0.004, *p* = 0.647; “job involves heavy manual or physical work”: Egger intercept = -0.014, SE = 0.028, *p* = 0.622; income: Egger intercept = -0.001, SE = 0.023, *p* = 0.975; “Townsend deprivation index at recruitment”: Egger intercept = 0.019, SE = 0.036, *p* = 0.611; “leisure/social activities: pub or social club”: Egger intercept = -0.007, SE = 0.043, *p* = 0.871; “leisure/social activities: sports club or gym”: Egger intercept = 0.004, SE = 0.121, *p* = 0.972). Further, as shown in Table 2 and Table 3, our MR-PRESSO analyses identify pleiotropic outlying SNPs and determine outlier-adjusted estimates after removing these outlying SNPs (“average total household income before tax”: PRESSO-adjusted OR 1.402, 95%CI 0.898–2.189, *p* = 0.137; “leisure/social activities: pub or social club”: PRESSO-adjusted OR 0.524, 0.023–11.765, *p* = 0.684). Remarkably, after PRESSO adjustments, we did not observe significant heterogeneity in the results for causal effects of income (Q = 38.149, I^2^ = 21.361%, *p* = 0.146) and pub or club (Q = 12.520, I^2^ = 36.102%, *p* = 0.069) on FGP risk. In addition, the results of effect estimates (β values) for the six socioeconomic traits on FGP risk in UVMR analyses are visualized in six scatter plots (Supplementary Figs. 7–12). Moreover, the results of effect estimates for individual SNPs in UVMR analyses are illustrated in six forest plots (Supplementary Figs. 13–18), and that for all SNPs are demonstrated in a summarized forest plot (Fig. 2).

**Table 3:** Heterogeneity and pleiotropy evaluations for genetically causal associations of socioeconomic traits with FGP risk

Socioeconomic traits	nSNP	Heterogeneity				Pleiotropy		
		Q	I^2^ (%)	*p*		Intercept	SE	*p*
Educational attainment	320	293.38	8.7	0.845		0.002	0.004	0.647
Job involves heavy manual or physical work	15	8.887	57.5	0.838		< 0.001	0.029	0.998
Average total household income before tax	32	47.818	35.2	0.027		0.001	0.023	0.975
PPRESSO adjustment	31	38.149	21.4	0.146		0.008	0.021	0.688
Type of accommodation lived in: a flat, maisonette or apartment	1	NA	NA	NA		NA	NA	NA
Type of accommodation lived in: a house or bungalow	1	NA	NA	NA		NA	NA	NA
Townsend deprivation index at recruitment	13	15.273	21.4	0.227		0.019	0.036	0.611
Leisure/social activities: pub or social club	10	21.437	58	0.011		0.007	0.043	0.871
PPRESSO adjustment	8	12.52	36.1	0.069		0.026	0.04	0.545
Leisure/social activities: sports club or gym	7	11.688	48.7	0.069		0.004	0.121	0.972

Abbreviations: CI = confidence interval; IVW = inverse-variance weighted; MR = Mendelian randomization; OR = odds ratio; SE = standard error; nSNP = number of single-nucleotide polymorphism; WM = weighted median

### MVMR results

#### Eligible SNPs

Next, we leveraged the SNPs surrogating for these six socioeconomic traits to establish the following two models to conduct MVMR analyses, depending on different combination patterns for the six socioeconomic traits: (1) Model 1: “EA”, “heavy physical work”, “income “, “TDI”, and “pub or club”; (2) Model 2: “EA”, “heavy physical work”, “income “, “TDI”, and “sports”. We clustered 402 SNPs for Model 1 and 408 SNPs for Model 2 respectively, and used them as integrated proxy instruments to conduct MVMR analyses.

#### LASSO feature selection

Additionally, we performed LASSO feature selection for the two MVMR models using the “mv_lasso_feature_selection” function, and removed “income” due to its severe collinearity with “heavy physical work” in Model 1. Accordingly, the remaining four traits were retained. Dissimilarly, none of the five traits was detected to carry collinearity in Model 2.

### Instrumental strength of MVMR Models

First, we calculated the proportion of phenotypic variances that were explained by our two clusters of SNPs and revealed that 402 SNPs as IVs for MVMR Model 1 explained 0.690% of phenotypic variances for EA, 0.702% for heavy physical work, 0.142% for TDI, and 0.117% for pub or club, respectively. As far as MVMR Model 2 was concerned, the proportion (i.e., R^2^-value) of phenotypic variations that were explained by our 408 integrated SNPs was 0.702% for EA, 0.718% for heavy physical work, 0.421% for income, 0.143% for TDI, and 0.108% for sports, respectively. Furthermore, we determined F-statistics symbolizing the total instrumental strength of our 402 SNPs (Model 1) and 408 SNPs (Model 2). Specifically, the former was respectively 2795.203, 650.161, 1147.773, and 1145.337 for the four traits, while the latter was 2753.746, 640.479, 969.778, 1130.869, and 1128.580 for the five traits. In summary, our findings supported strong, robust, and reliable genetic proxies for socioeconomic traits to investigate their causal associations with FGP risk.

#### Effect estimations based on model 1

**Fig. 3:**
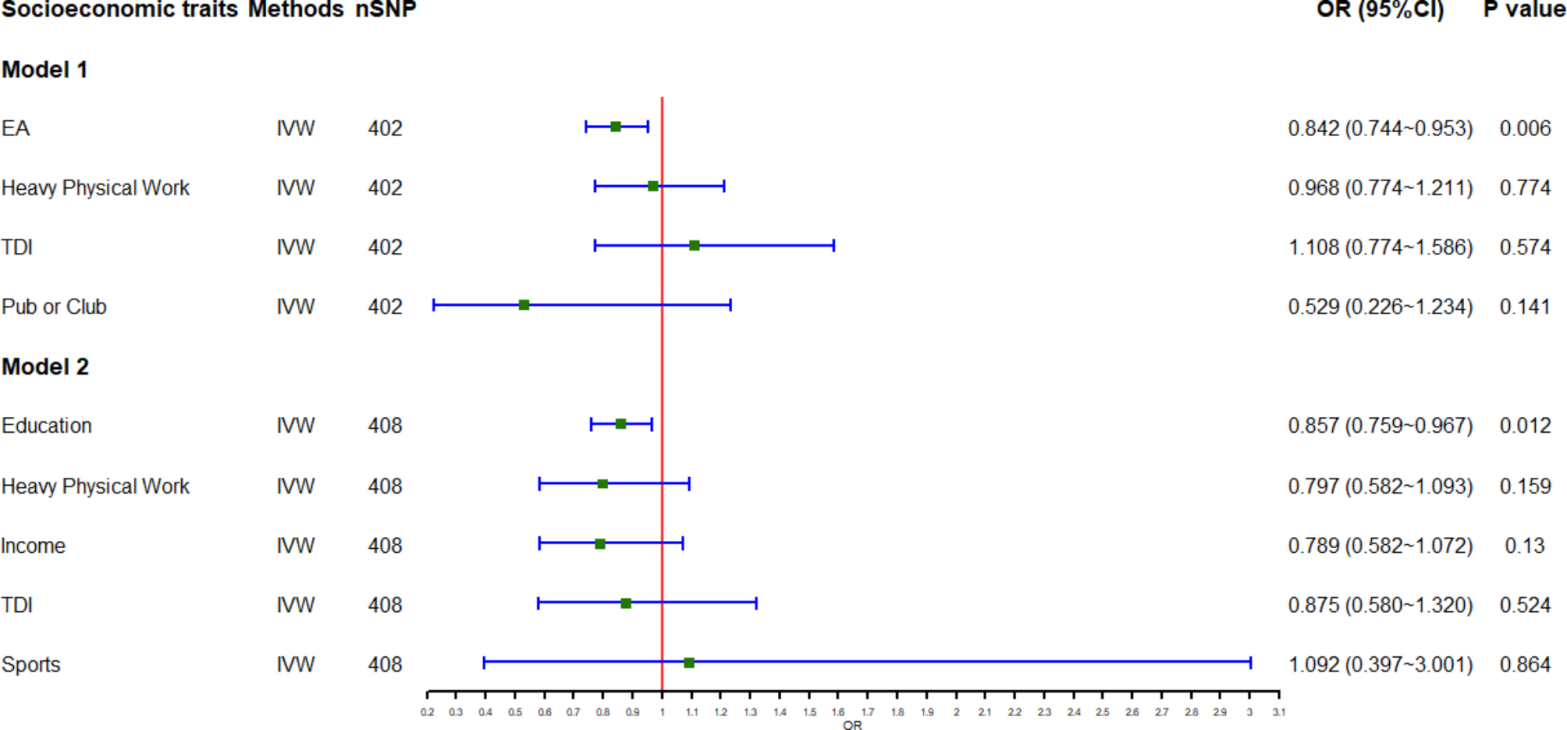
**Forrest plot for causal associations of socioeconomic traits with FGP risk based on IVW MVMR Model 1 and 2.** The forest plot demonstrates that EA is independently and predominantly responsible for causal effects of socioeconomic traits on FGP risk after adjusting for heavy physical work, TDI, and pub or club (Model 1), or adjusting for heavy physical work, income, TDI, and sports (Model 2). **Abbreviations**: nSNP = number of single-nucleotide polymorphism; OR = odds ratio; CI = confidence interval; EA = educational attainment; FGP = female genital prolapse; heavy physical work = job involves heavy manual or physical work; income = average total household income before tax; IVW = inverse-variance weighted; MR = Mendelian randomization; MVMR = multivariable Mendelian randomization; pub or club = leisure/social activities: pub or social club; sports = leisure/social activities: sports club or gym; TDI = Townsend deprivation index at recruitment

The results of our MVMR analyses based on Model 1 are displayed in Fig. 3; Table 4. As the figure and table indicate, higher EA (OR 0.842, 95%CI 0.744–0.953, *p* = 0.006) has protective effect on FGP risk, and predominantly accounts for the associations between the four socioeconomic traits and FGP risk after adjusting for “heavy physical work” (OR 0.968, 95%CI 0.774–1.211, *p* = 0.774, TDI (OR 1.108, 0.774–1.586, *p* = 0.574), and “pub or club” (OR 0.529, 0.226–1.234, *p* = 0.141). In other words, the women who achieve higher EA have one-seventh lower predisposition to incident FGP than those own lower EA, regardless of the involvement of heavy physical work in their jobs, TDI, and leisure activities such as attendance to social pub or club.

**Table 4:** Causal effects of multiple socioeconomic traits on FGP risk based on IVW MVMR model

Socioeconomic traits	nSNP	β	SE	OR	95%CI	*p*
Model 1						
EA	402	-0.248	0.091	0.842	0.744–0.953	0.006
Heavy physical work	402	-0.047	0.165	0.968	0.774–1.211	0.774
TDI	402	0.148	0.264	1.108	0.774–1.586	0.574
Pub or Club	402	-0.92	0.624	0.529	0.226–1.234	0.141
**Model 2**						
EA	408	-0.223	0.089	0.857	0.759–0.967	0.012
Heavy Physical Work	408	-0.327	0.232	0.797	0.582–1.093	0.159
Income	408	-0.341	0.225	0.789	0.582–1.072	0.13
TDI	408	-0.193	0.303	0.875	0.580–1.320	0.524
Sports	408	0.127	0.744	1.092	0.397–3.001	0.864
**Abbreviations**: FGP = female genital prolapse; IVW = inverse-variance weighted; MVMR = Multivariable Mendelian randomization; SE = standard error; OR = odds ratio; CI = confidence interval; EA = Educational attainment; Heavy physical work = job involves heavy manual or physical work; Average total household income before tax; nSNP = number of single-nucleotide polymorphism; Pub or club = Leisure/social activities: pub or social club; Sports = Leisure/social activities: sports club or gym; TDI = Townsend deprivation index at recruitment.						

#### Effect estimation based on model 2

The results of our MVMR analyses based on Model 2 are also exhibited in Fig. 3; Table 4. Similar to the MVMR findings acquired from Model 1, our MVMR results from Model 2 also indicate that higher EA (OR 0.857, 95%CI 0.759–0.967, *p* = 0.012) has protective effect on FGP risk, and plays a predominant role in the associations of the five socioeconomic traits with FGP risk, independent of heavy physical work (OR 0.797, 0.582–1.093, *p* = 0.1590), income (OR 0.789, 0.582–1.072, *p* = 0.130), TDI (OR 0.875, 0.580–1.320, *p* = 0.524), and sports (OR 1.092, 0.397–3.001, *p* = 0.864). That is to say, the women with higher EA are one-quarter less likely to have incident FGP than those with lower EA, regardless of the involvement of heavy physical work in their jobs, income, TDI, and engagement in such leisure activities as sports.

### Statistical power

The results of statistical power for the nine groups of integrated SNPs in estimating causal associations of socioeconomic traits with FGP risk are presented in Table 2. As the table manifests, the statistical power of all our nine groups of SNPs is suboptimal but moderate.

## Discussion

### POP prevalence across socioeconomic populations

There have been a variety of characteristics used as indicators measuring socioeconomic status in health research [61, 62], including educational attainment [61, 63, 64], occupational nature [64, 65], income level [61, 63, 64], poverty degree proxied by TDI [63, 65], leisure activities [66–69], and accommodational conditions [70, 71]. Torneto et al. [72] observed a POP prevalence of 0 in high-socioeconomic group, 21% in middle-socioeconomic group, and 45% in low-socioeconomic group. It was presumed that the significant differences concerning POP prevalence among different socioeconomic populations might be attributed to diverse genital hygiene, different nutritional status, and/ or different knowledge about this condition.

### Main findings

#### UVMR results

Our UVMR analyses based on IVW method revealed a causal association of lower EA with higher FGP risk, suggesting that women with lower EA are more susceptible to have FGP than those with the opposite features. Moreover, our UVMR results are robust in consideration of no heterogeneity or pleiotropy in effect estimates. Besides, our results of reverse directional UVMR analyses expelled the possibility of reverse causality between FGP and any of the eight socioeconomic traits, which had been regarded as one of the major methodological limitations in previous observational studies.

#### MVMR results

Our MVMR analyses further uncovered that among the four socioeconomic traits (in MVMR Model 1) and five socioeconomic traits (in MVMR Model 2), there was only one trait, namely, EA that independently and predominantly accounted for the causal associations of these socioeconomic traits with FGP risk.

### Potential mechanisms

We speculated that higher educational attainment is associated with a lower probability of heavy physical work [73], higher income [74], better nutrition [75], more access to better healthcare services [76, 77], routine medical examinations, lower probability of early marriage [78, 79] and vaginal delivery [80]. In previous studies, all the above factors have an impact on FGP risk. Also, it has been well established that socioeconomic factors, such as education, strongly shape health-related behaviors [62], potentially due to superior self-management and healthcare engagement practices afforded to those with greater education, suggesting that individuals with higher educational attainment may be more inclined to pay attention to their health and take preventive measures to maintain good health, which could decrease the risk of FGP.

### Comparisons to previous studies

#### Educational attainment

Although it has been frequently found in previous observational studies that the decreased prevalence of POP is significantly associated with increased level of education by univariate analysis, there has been a controversy with regard to independent association of EA with the prevalence of POP. Some multivariate logistic regression analyses consistently evidenced that absent or insufficient formal education was an independent risk factor of having FGP or POP [6–9]. Given the independent association of insufficient knowledge about POP with low EA that was reported in O’Neill’s [81] and Chen’s [82] studies, promoting girls’ education was recommended to possibly decline the prevalence of POP. However, the independent association was not observed in Wang’s [15] retrospective cross-sectional study and Lovejoy’s [16] prospective cohort study. Explicitly, our UVMR results have resolved the controversy existing in previous observational studies, and clarified that higher EA is causally associated with lower FGP risk, while “job involving heavy physical work” has no causal effect on higher FGP risk. Furthermore, our MVMR analyses provide genetic evidence supporting EA as the independent and predominant trait that accounts for the relationship between multiple socioeconomic traits and FGP risk.

#### Strenuous work

Jobs needing heavy physical labor, such as heavy load carrying, are ubiquitous activities for women living in low-income countries. In previous observational studies, there have been existing discrepant findings regarding whether strenuous work is independently associated with prevalence of POP. Some studies concluded that strenuous work [9–13] was an independent determinant for having POP *via* multivariate logistic regression analyses. Therefore, some researchers recommended avoidance of carrying heavy objects for attenuating POP risk. In contrast, Devkota’s [18] multivariate analysis did not agree on such a significant association between heavy load carrying and uterine prolapse (UP). Our UVMR and MVMR analyses have resolved this ambiguity and clearly denied any causal effect of “job involving heavy manual or physical work” on FGP risk.

#### Income

UVP, the main constituent of FGP, is a major cause of mortality and morbidity among women in low-income countries such as Nepal [83]. Woodman et al.’s multivariate logistic analysis [14] established that annual income of $10,000 or less in women was independently associated with more severe POP, relative to that of over $10,000 (*p* < 0.001). The mechanism underlying the associations between income level and FGP risk in observational studies remains unexplained, which can be interpreted partially by previous findings that the women with lower income are more predisposed toward a higher probability of early marriage [84–86], vaginal delivery [87–91], and strenuous work [13, 92, 93].

#### Townsend deprivation index

The TDI is defined as an area-based measure of socioeconomic deprivation and regarded as a proxy of individuals’ socioeconomic deprivation, with a higher TDI referring to a higher level of socioeconomic deprivation [65]. Also, there has been insufficient evidence from observation studies examining the association of TDI with the prevalence of POP. Unambiguously, our UVMR and MVMR analyses ascertained that TDI had no causal effect on risk of FGP.

#### Leisure or social activities

Scientists have long known that the socioeconomic conditions in which children grow up impact their health behaviors in adulthood —particularly physical activity [64]. Participation in active free play, namely leisure activities, decreases in girls from low-income residential areas relative to their counterparts from high-income ones [64]. In addition, compared with the girls from high socioeconomic backgrounds, those from low socioeconomic backgrounds usually report a lower preference for physical activity [64, 94, 95]. In previous observational studies, there has been a lack of definite evidence supporting an independent association of leisure or social activities with odds of FGP. In a multivariate logistic regression analysis by Nygaard et al. [17], leisure activity was not found to be independently correlated to probability of POP. Our MVMR analyses, for the first time, disentangled the ambiguity deposited in previous observational studies, and definitely advocated that “leisure or social activities” had no causal effect on FGP risk, let alone independent and predominant role in the associations of socioeconomic traits with FGP risk.

#### Interpretation for discrepant findings

Based on the complexity of the causal pathways resulting from social factors [96], such as education, previous observational studies have found that the correlation between other socioeconomic factors and FGP may be influenced by different levels of education. In addition, educational attainment is a reliable proxy for measuring socioeconomic status as it is determined early in life and is strongly associated with later life measures of socioeconomic position, such as employment and income [97]. In contrast, individual-level proxies such as income are prone to reporting bias and ecological fallacy [98]. Therefore, educational attainment is a valuable indicator for investigating the role of socioeconomic factors in FGP. Furthermore, the discrepancies between the results of observational studies and MR analyses can be attributed to the limitations of observational studies and the inherent superiority of MR analyses. Unlike observational studies, MR analyses can overcome the issues of confounding and reverse causality by integrating a set of SNPs that are strongly associated with multiple socioeconomic traits in a same model. This enables MR analyses to simultaneously estimate potential causal effects of multiple socioeconomic traits on one outcome event, i.e., FGP. Therefore, MR analyses provide a more robust and reliable approach to identifying causal relationships between socioeconomic factors and FGP than observational studies.

### Public health implications

Our UVMR and MVMR findings have important implications for public health by providing new insights into the pathogenesis underlying female genital prolapse (FGP) from a genetic perspective. Our study results add to the existing research on the association between FGP and socioeconomic status and suggest that modifying specific socioeconomic traits, such as promoting education among girls, may serve as a potential prophylactic measure against FGP. It is essential to extend educational attainment and initiate public health education programs, especially in countries where education is not widely available, as this can have a long-term impact on preventing FGP beyond simply raising people’s incomes. While it may be challenging to change educational attainment in adulthood, screening for FGP should be prioritized among women with lower educational attainment from a preventive perspective and early diagnosis standpoint.

### Strengths in our study

There are several strengths in the present study. To the best of our knowledge, our study is the first MR study focused on causal associations of multiple socioeconomic traits with FGP risk using large-scale GWAS data. Undoubtedly, our UVMR and MVMR analyses are superior over previous observational studies because we extract summary data from GWASs with a much larger sample size and a huge number of SNPs. Moreover, the results are robust and reliable by virtue of no heterogeneity or pleiotropic effect. Extremely importantly, our two MVMR models prove to be strong, robust, and reliable genetic proxies for socioeconomic traits.

### Limitations in our study

We confess several limitations in our study. Above all, although FGP predominates in female POP population, FGP is not exactly equal to POP. Owing to no GWAS available for POP, we had to resort to FGP as an outcome event instead of POP. It is not sound enough to implement comparisons of conclusions regarding associations of socioeconomic traits with two different, even if approximate, outcome events between observational studies and MR analyses. Secondly, a weak statistical power of our SNPs surrogating compromises the precision and reliability in estimating causal effect on FGP risk. Therefore, our conclusions should be used with caution. The unfavorable statistical power will not be improved unless the advent of updated GWAS datasets with a larger sample size and more eligible SNPs representing socioeconomic traits and FGP in the future. After all, we acknowledge that this study provides insights into the causal effect of EA on POP from genetic perspective. Still, the underlying mechanisms behind this causal relationship remain elusive and are worth further exploration in, perhaps, a mediation effect analysis of heavy physical work, income, TDI, and social activities, or in a pathway analysis involving molecular mechanisms and hormonic factors in the future.

## Conclusion

To sum up, our UVMR and MVMR analyses provided genetic evidence that as a representative of socioeconomic traits, educational attainment is causally associated with risk of female genital prolapse, and even independently and predominantly accounts for the association of socioeconomic status with risk of female genital prolapse.

## Electronic supplementary material

Below is the link to the electronic supplementary material.

Supplementary Material 1

Supplementary Material 2

Supplementary Material 3

Supplementary Material 4

Supplementary Material 5

Supplementary Material 6

Supplementary Material 7

Supplementary Material 8

Supplementary Material 9

Supplementary Material 10

Supplementary Material 11

Supplementary Material 12

Supplementary Material 13

Supplementary Material 14

Supplementary Material 15

Supplementary Material 16

Supplementary Material 17

Supplementary Material 18

Supplementary Material 19

## Data Availability

The datasets analyzed in this study are publicly available summary statistics. Summary statistics for the GWASs concerning the exposures and outcome are available from the IEU GWAS database (https://gwas.mrcieu.ac.uk/). For the datasets used and/or analyzed, and the codes used during the current study, please contact the corresponding author at chg1207879340@163.com (Honggu Chen) on reasonable request.

## Abbreviations

FGP: Female genital prolapse
POP: Pelvic organ prolapse
GV: Genetic variants
EA: Educational attainment
TDI: Townsend deprivation index at recruitment
GWAS: Genome-wide association study
SNP: Single nucleotide polymorphism
UVMR: Univariate mendelian randomization
MVMR: Multivariable mendelian randomization
IV: Instrumental instrument
IVW: Inverse variance weighted
WM: Weighted median
MR-PRESSO: MR-pleiotropy residual sum and outlier
CI: confidence interval
MAF: Minor allele frequency
