# Supplementary material for: Evaluation for causal effects of socioeconomic traits on risk of female genital prolapse (FGP): a multivariable Mendelian randomization analysis
Source: BMC Med Genomics. 2023 Jun 9;16:125. doi: 10.1186/s12920-023-01560-5 (PMC10251634; doi:10.1186/s12920-023-01560-5)
Supplement: Supplementary file 6 — Supplementary Material 6 [file 12920_2023_1560_MOESM6_ESM.docx]

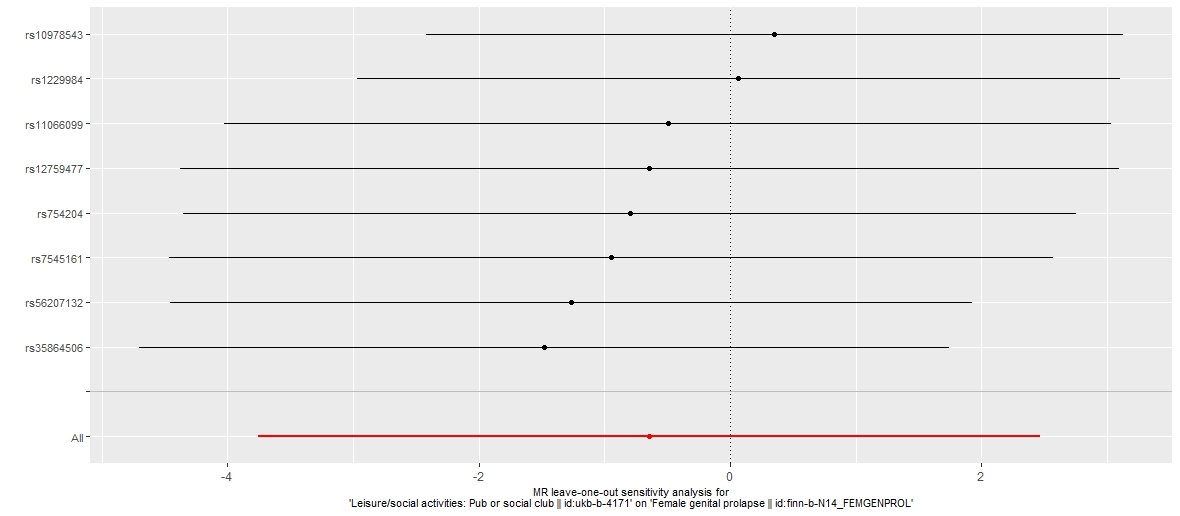


**Supplementary Figure 5. Leave-one-out plot for sensitivity analysis of single SNP effect on pub-to-FGP UVMR results.**

Leave-one-out plot using IVW method by sequentially re-evaluating the causal estimate after discarding one SNP at a time, which helps determine whether the overall effect is driven by the specific genetic variant. The black point denotes the causal effect estimate of pub on FGP after discarding a certain SNP, and the black line signifies the 95% CI of estimate. The red point symbolizes the causal effect estimate of pub on FGP with the eight valid SNPs, and the red line indicates the 95% CI of the estimate. **Abbreviations:** FGP = female genital prolapse; SNP = number of single-nucleotide polymorphism; UVMR = univariate Mendelian randomization; sport= leisure/social activities: sports club or gym; CI = confidence interval; pub=Leisure/social activities: pub or social club.
