# Supplementary material for: Evaluation for causal effects of socioeconomic traits on risk of female genital prolapse (FGP): a multivariable Mendelian randomization analysis
Source: BMC Med Genomics. 2023 Jun 9;16:125. doi: 10.1186/s12920-023-01560-5 (PMC10251634; doi:10.1186/s12920-023-01560-5)
Supplement: Supplementary file 10 — Supplementary Material 10 [file 12920_2023_1560_MOESM10_ESM.docx]

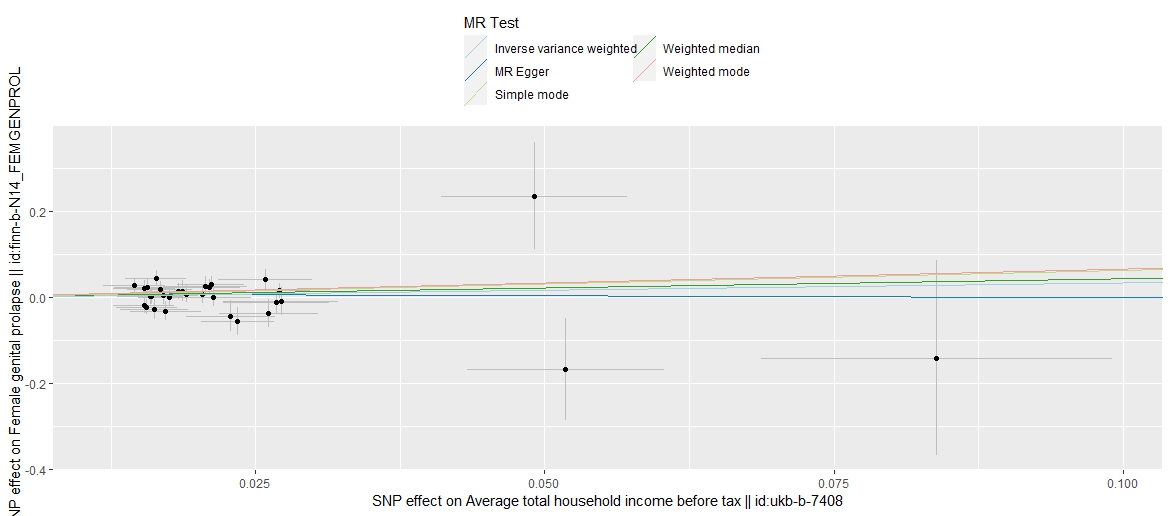


**Supplementary Figure 9. Scatter plot for UVMR analysis of causal relationship between "income" and FGP risk using five MR methods.**

The β value with SE is plotted to demonstrate effect estimate of each SNP (*n* = 31) for causal association of "income" (x-axis) with FGP (y-axis). The slope of each line represents the UVMR estimate (β value) for the individual SNP. Error bar represents SE of effect size. **Abbreviations:** FGP = female genital prolapse; MR = Mendelian randomization; SE = standard error; SNP = number of single-nucleotide polymorphism; UVMR = univariate Mendelian randomization; income = average total household income before tax.
