# Supplementary material for: Evaluation for causal effects of socioeconomic traits on risk of female genital prolapse (FGP): a multivariable Mendelian randomization analysis
Source: BMC Med Genomics. 2023 Jun 9;16:125. doi: 10.1186/s12920-023-01560-5 (PMC10251634; doi:10.1186/s12920-023-01560-5)
Supplement: Supplementary file 15 — Supplementary Material 15 [file 12920_2023_1560_MOESM15_ESM.docx]

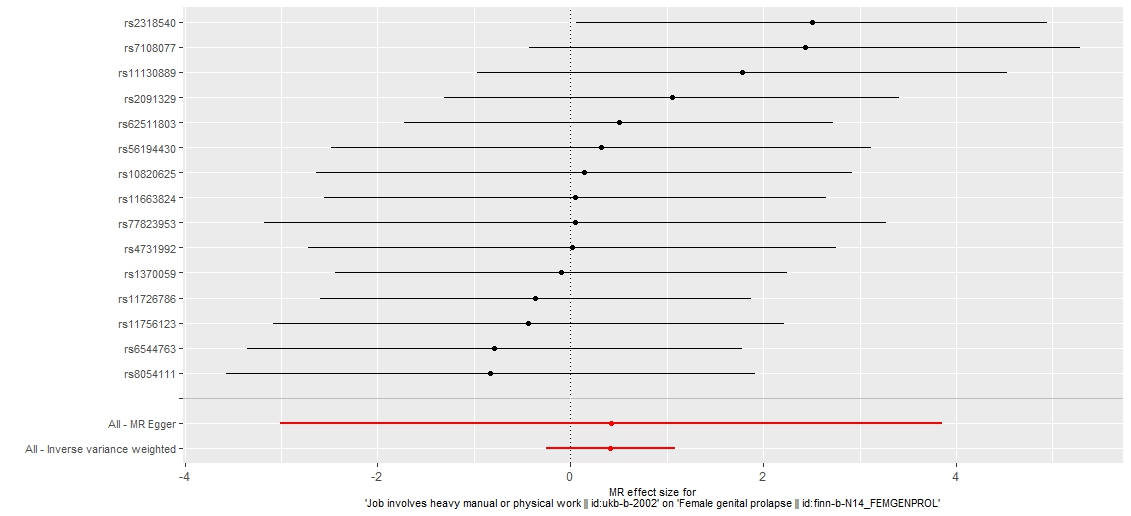


**Supplementary Figure 14. Forest plot for UVMR analysis of single and summarized SNPs effects on relationship between heavy physical work and FGP risk with 15 individual SNPs.**

A black point denotes the effect estimate of heavy physical work on FGP using a single SNP, and the black line signifies the 95% CI of the estimate. The red point symbolizes overall effect estimate of heavy physical work on FGP with 15 SNPs using the Egger and IVW method, and the red line indicates the 95% CI of the estimate. **Abbreviations:** FGP = female genital prolapse; SNP = number of single-nucleotide polymorphism; UVMR = univariate Mendelian randomization; CI = confidence interval; heavy physical work = Job involves heavy manual or physical work.
