# Supplementary material for: Evaluation for causal effects of socioeconomic traits on risk of female genital prolapse (FGP): a multivariable Mendelian randomization analysis
Source: BMC Med Genomics. 2023 Jun 9;16:125. doi: 10.1186/s12920-023-01560-5 (PMC10251634; doi:10.1186/s12920-023-01560-5)
Supplement: Supplementary file 17 — Supplementary Material 17 [file 12920_2023_1560_MOESM17_ESM.docx]

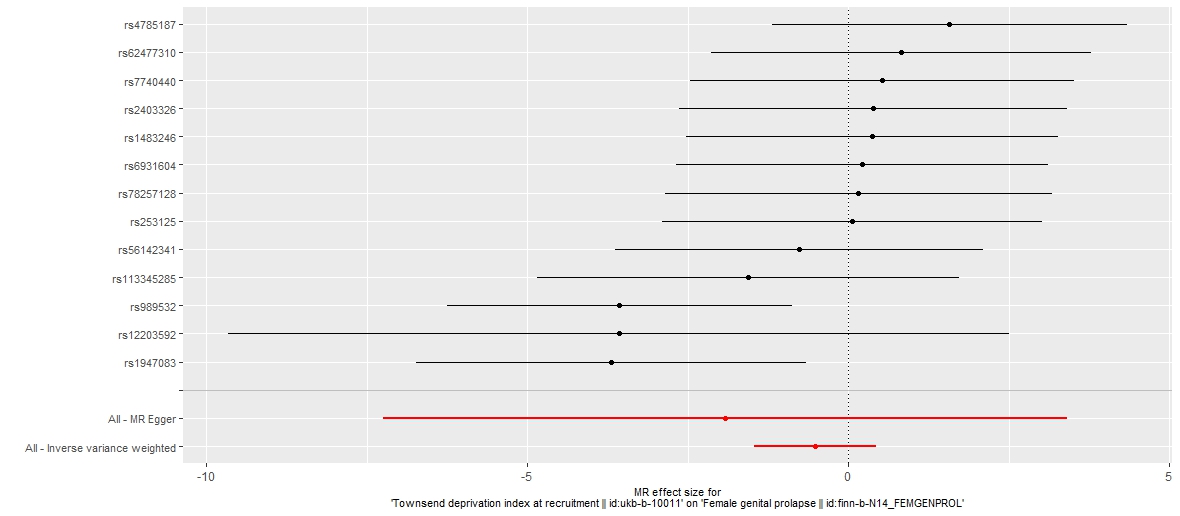


**Supplementary Figure 16. Forest plot for UVMR analysis of single and summarized SNPs effects on relationship between TDI and FGP risk with 13 individual SNPs.**

A black point denotes the effect estimate of TDI FGP using a single SNP, and the black line signifies the 95% CI of the estimate. The red point symbolizes overall effect estimate of TC on FGP with 8 SNPs using the Egger and IVW method, and the red line indicates the 95% CI of the estimate. **Abbreviations:** FGP = female genital prolapse; SNP = number of single-nucleotide polymorphism; UVMR = univariate Mendelian randomization; TDI = Townsend deprivation index CI = confidence interval.
